# Modulation of the Gut Microbiota Structure and Function by Two Structurally Different Lemon Pectins

**DOI:** 10.3390/foods11233877

**Published:** 2022-12-01

**Authors:** Jenni Firrman, Karley Mahalak, Jamshed Bobokalonov, LinShu Liu, Jung-Jin Lee, Kyle Bittinger, Lisa M. Mattei, Rizalina Gadaingan, Adrienne B. Narrowe, Johanna M. S. Lemons

**Affiliations:** 1 United States Department of Agriculture, Agriculture Research Service, Eastern Regional Research Center, 600 East Mermaid Lane, Wyndmoor, PA 19462, USA; 2 Children’s Hospital of Philadelphia, Division of Gastroenterology, Hepatology, and Nutrition, 3401 Civic Center Blvd., Philadelphia, PA 19104, USA

**Keywords:** gut microbiota, pectin, lemon pectin, molecular weight, degree of esterification, short-chain fatty acids, *Lachnospiraceae*

## Abstract

Pectins are plant polysaccharides consumed as part of a diet containing fruits and vegetables. Inside the gastrointestinal tract, pectin cannot be metabolized by the mammalian cells but is fermented by the gut microbiota in the colon with the subsequent release of end products including short-chain fatty acids (SCFA). The prebiotic effects of pectin have been previously evaluated but reports are inconsistent, most likely due to differences in the pectin chemical structure which can vary by molecular weight (MW) and degree of esterification (DE). Here, the effects of two different MW lemon pectins with varying DEs on the gut microbiota of two donors were evaluated in vitro. The results demonstrated that low MW, high DE lemon pectin (LMW-HDE) altered community structure in a donor-dependent manner, whereas high MW, low DE lemon pectin (HMW-LDE) increased taxa within *Lachnospiraceae* in both donors. LMW-HDE and HMW-LDE lemon pectins both increased total SCFAs (1.49- and 1.46-fold, respectively) and increased acetic acid by 1.64-fold. Additionally, LMW-HDE lemon pectin led to an average 1.41-fold increase in butanoic acid. Together, these data provide valuable information linking chemical structure of pectin to its effect on the gut microbiota structure and function, which is important to understanding its prebiotic potential.

## 1. Introduction

Pectin is a heteropolysaccharide widely distributed throughout the plant cell wall and middle lamella [1,2]. It is structurally complex and comprised of O-1 and O-4 linked galacturonic acids (GalA) with three major units, homogalacturonan (HG), rhamnogalacturonan I (RG-I), and rhamnogalacturonan II (RG-II) [1,2,3]. The pectin molecule itself can be broadly categorized based on two key parameters, molecular weight (5–2, 180 kDa), and degree of esterification (DE), also referred to as degree of methoxylation (DM), which is the percent composition of methyl esterified GalA compared to GalA [2,4,5]. A pectin molecule with a DE of less than 50% is considered low degree of esterification pectin (LDE) and one with a DE of greater than 50% is considered high degree of esterification pectin (HDE) [2]. The structure of pectin is highly variable, and differs between plants and developmental phases, such as fruit ripening [2,3].

Pectin is consumed by humans as part of a diet containing fruits and vegetables [5]. In the gastrointestinal tract human enzymes are unable to break down pectin’s glycosidic links [5,6]. Therefore, pectin will enter the colon and come into contact with the resident gut microbiota, a complex community of microorganisms that includes a robust bacterial component that plays a well-known role in human health [5,6,7]. Within the colon, the gut microbiota is known to play a role in protecting the host from colonization of pathogens through nutrient and physical competition, synthesizes vitamins, and produces an array of metabolites, such as short chain fatty acid (SCFA), that can modulate the immune system [7]. The gut microbiota is able to ferment pectin because the microbes carry polysaccharide utilization loci (PULs) and release carbohydrate-active enzymes that act in concert to break down the molecule and allow pectin to be used as a carbon source with the subsequent release of the end-product metabolites SCFAs [7,8]. *Bacteroides* species are thought to be the primary pectin degraders within the community and in particular, *Bacteroides thetaiotamicron* has been previously found to carry the PULs capable of degrading the pectin structure [8,9]. Taxa within the family *Lachnospiraceae* also contain pectin degrading enzymes, i.e., hydrolases, lyases, and esterases, but to a lesser extent [10]. It has also been reported that other, lower abundant taxa, such as *Faecalbacterium prausnitzii* and *Eubacterium eligens* contribute to pectin fermentation [11].

Pectin is often classified as a prebiotic, namely a substrate that is not digested by human factors but is fermented by the gut microbiota, stimulating growth of beneficial taxa and enhancing microbial activities to produce a health benefit [9,10,12]. Administration of pectin as a prebiotic has been previously associated with a number of positive health outcomes, such as reduction of inflammation, immunomodulation, inhibition of obesity, and increased cellular barrier function [5,12,13,14]. However, due to the complexity of the gut microbiota, which includes multifactorial cross-reactions and cross-feeding between taxa, there is still a lot to learn about how pectin influences human health via gut microbiome modulations. Pectin size inhibits direct cellular uptake by the microbes, and so the pectin backbone must be depolymerized first and then the side chains must be removed, converting the structure into smaller oligosaccharides, that can be potentially used in a cross-feeding strategy [6]. Furthermore, the process of pectin metabolism by the gut microbiota is variable and depends on the chemical properties of the pectin in question [15,16,17]. Of particular importance are the DE and MW, which directly affect pectin intestinal fermentability [16,17]. 

Several previous studies have reported the prebiotic effects of different types of pectin with varying DE and MW. It has been reported that pectins with a smaller MW had a higher rate of fermentation and increased SCFA yield [18], and the reduction of MW in artichoke and citrus pectins promoted the growth of *Bifidobacterium* and *Lactobacillus* [19]. A previous in vitro study looking at citrus pectins and sugar beet pectin noted that DE was the most important parameter for pectin modification of the gut microbiota [16]. This study also reported that pectin with a high DE generated more propionate and SCFAs compared to low DE, whereas a low DE pectin stimulated levels of *Bifidobacterium* [16,20]. In vivo and in vitro studies have found that low DE citrus pectins are fermented more efficiently than high DE pectins [21]. Conversely, for artichoke and sunflower pectins it has been previously reported that MW and DE had no impact on SCFA production [19]. To date, results on pectin’s modulation of the gut microbiota have been inconsistent, most likely due to differences in the chemical structures of the pectin used, as different forms of pectin may produce varying results [9]. 

In this study we set out to understand how the MW and DE effect the prebiotic function of citrus pectin on the gut microbiota. To isolate the interactions between pectin and the microbes, an in vitro experimental design was utilized to remove the multifarious interactions that occur between the gut microbiota and mammalian cells in an in vivo model [22]. Changes to the gut microbiota community structure and function were determined using 16S rRNA gene sequencing and SCFA analysis [22,23]. The results of this study provided detailed information on how pectins with different chemical structures modulate the gut microbiota in divergent manners, which advances our understanding of how pectin MW and DE may impact this interaction, which is critical to its application as a prebiotic. 

## 2. Materials and Methods

### 2.1. In Vitro Cultivation of the Gut Microbiota and Experimental Design

A set of four Eppendorf Bioflow 320 bioreactors was used to cultivate the gut microbiota of two adult donors in duplicate (Supplementary Figure S1). The inocula were purchased from the company Openbiome (Sommerville, MA, USA) as fecal homogenates from random adult donors as described previously [22]. The inocula were characterized via 16S rRNA amplicon sequencing to determine the initial community structure (Supplementary Figure S2). The communities were maintained as described previously using Defined Media (DM), purchased from the company ProDigest^®^ (Gent, Belgium), as a source of nutrition and Pancreatic Juice (PJ) as a source of pancreatic and biliary enzymes [22]. During the treatment phase, the DM was supplemented with 1% lemon pectin. Lemon pectin was selected for this study due to reports of its prebiotic potential and because it can be recovered from lemon waste produced during juice-processing [24,25,26]. The lemon pectin was obtained from CP Kelco, Inc (Atlanta, GA, USA), and characterized using previously described methods (Supplementary Table S1) [27]. One lemon pectin with a molecular weight of 308 kDa and 31% DE was termed HMW-LDE, and the second lemon pectin with a molecular weight of 122 kDa and 66% DE was termed LMW-HDE.

Prior to inoculation, each bioreactor was filled with 700 mL of DM and 300 mL of PJ [22]. The fecal homogenates were thawed according to the supplier’s guidelines and each bioreactor was inoculated with a 5% volume of homogenate. Two bioreactors were inoculated with homogenate from donor 1 and two bioreactors were inoculated with homogenate from donor 2. The gut microbiota community within each bioreactor was maintained by feeding three times a day (every 8 h) with a 200 mL volume of 70% DM and 30% PJ as previously described [28,29]. The communities were given time to reach stability (>2 weeks) [22], and then samples harvested over the course of 4 days as the control period. Following the control period, the DM was replaced with DM containing 1% lemon pectin for 3 days as the treatment period of the experiment. 

During lemon pectin treatment, one bioreactor from each donor was provided DM containing HMW-LDE lemons pectin and one bioreactor from each donor was provided DM containing LMW-HDE lemon pectin. After the treatment period, all bioreactors were supplied DM without lemon pectin for 9 days in the post-treatment period. Three samples from the control, lemon pectin treatment, and post-treatment period were harvested and used for analysis (Supplementary Figure S1). For all figures, except the principal coordinate analyses, the results indicate the average of the 3 samples and their standard deviation. For DNA analysis, a 1 mL aliquot of culture was harvested, and for SCFA analysis, a 5 mL aliquot of culture was harvested. All samples were centrifuged at 5000× *g* for 10 min at 4 °C. For DNA, the supernatant was discarded, and the bacterial pellet saved. For SCFA analysis, the supernatant was passed through a 0.22 µM filter (Whatman) and saved. After harvesting, all samples were stored at −80 °C until needed. 

### 2.2. S rRNA Sequencing

DNA was extracted from samples of communities during the control, lemon pectin treatment, and post-treatment period using the DNEasy Powersoil Kit as described previously (Qiagen, Hilden, Germany) [23,28]. The amount of DNA from each sample was quantified using Quant-iT PicoGreen dsDNA Assay Kit (Thermo Fisher Scientific, Waltham, MA, USA) and 16S rRNA gene sequencing was performed on the V1-V2 region using the Miseq platform (Illumina, San Diego, CA, USA) and a 2 × 250 bp chemistry as described previously [23,28]. Controls run included extraction blanks, DNA-free water, and a positive control of 8 artificial 16S gene fragments purchased from Integrated DNA Technologies (IDT, Coralville, IA, USA) [30]. 

### 2.3. Bioinformatic Analysis and Statistics

Data generated from the 16S rRNA V1-V2 region was processed and examined using the QIIME2 pipeline as described previously with DADA2 implemented [31,32,33]. The resulting amplicon sequence variants (ASVs) were identified using the Naïve-Bayes classifier that was trained on Green Genes database [34,35]. A rooted phylogenetic tree was first generated and then used to generate diversity and UniFrac distances. MAFFT was used to align multiple sequences and FastTree was used to generate the midpoint rooted tree [36,37]. Statistical differences in overall microbiome community similarity as determined by 16S rRNA V1-V2 region sequencing were assessed by the PERNAMOVA test, a non-parametric test of pairwise distance between samples [38]. When multiple comparisons were carried out, p-values were corrected to control for the false discovery rate using the method of Hochberg and Benjamini [39]. For alpha diversity, statistical changes were determined using a Single Factor ANOVA followed by a Student’s *t*-test and considered statistically significant when the *p*-values ≤ 0.05.

In order to detect significant shifts in the concentrations of each SCFA with the experimental conditions and by donor, for each of the four SCFAs quantified, we independently performed an ANOVA followed by Tukey’s Honestly Significant Difference post hoc test. Pairwise comparisons between full interaction terms having adjusted *p*-values ≤ 0.05 were considered significant. 

### 2.4. Detection of Total Bacteria and Bifidobacterium Using qPCR

Levels of *Bifidobacterium* were quantified as described previously using qPCR and the following primers: forward Bif243F 5′-TCGCGTCYGGTGTGAAAG-3′ and reverse Bif243R 5′-CCACATCCAGCRTCCAC-3′ [40]. Primers and a g-block for the standard curve were ordered from IDT. A Roche Lightcycler^®^ 96 instrument was used for this assay. The standard curve was generated by preparing 10× serial dilutions from 1 × 10^7^–1 × 10^2^ copies/µL. Each reaction contained 1 µL of extracted DNA diluted 100× in qPCR grade water (Roche, Basel, Switzerland), 500 nM each primer, 2× Applied Biosystems SYBR Green PCR Master Mix (Fisher Scientific, Waltham, MA) for a total of 20 µL. The following times and temperatures were used for the reaction: 95 °C for 5 m, followed by 40 cycles of 95 °C for 15 s, 58 °C for 20 s, and 72 °C for 30 s, and ended with 83 °C for 30 s, 94 °C for 15 s and a melting curve analysis. All samples were run in triplicate, negative controls were included. Roche Lightcycler^®^ software version 1.02.00.0086 was used to determine absolute levels of *Bifidobacterium*.

### 2.5. Short-Chain Fatty Acid Quantification

Short-chain fatty acids (SCFAs) were quantified using a GC/MS Shimadzu QP2010 Ultra (Shimadzu, Kyoto, Japan) equipped with Stabilwax-DA column, 30 m, 0.25 mm ID, 0.25 μm, (Restek Corporation, Belfonte, PA, USA) following a previously published protocol [23,28]. All reagents were obtained from Sigma Aldrich. Briefly, samples were centrifuged at 5000× *g* at 4 °C for 10 min then the supernatant was filtered with a 0.2 μM PES filter (Corning, Corning, NY, USA). Samples were prepared and loaded to GC autosampler and analyzed using a Gas Chromatography Mass Spectrophotometer (GC-MS) [23,28]. Sample at a volume of 1 µL was input as a 1:20 split mode at 260 °C, the interface temperature was 250 °C and ion source temperatures was 220 °C. An initial temperature of 125 °C was held for 1 m, then increased to 170 °C at a rate of 30 °C/m, then to 181.5 °C at a rate of 20 °C/m, held for 0.5 m, and then finally raised to 220 °C at a rate of 50 °C/min for 2 m. The following SCFAs were analyzed: acetic acid, propanoic acid, butanoic acid, pentanoic acid, hexanoic acid, 2-methylbutanoic acid, isovaleric acid, and isobutyric acid. Total SCFAs were calculated by summing all SCFAs detected; branch chained SCFAs (BCSCFAs) were calculated by summing all BCSCFAs detected. The average fold change in total SCFAs and acetic and butanoic acids was calculated by averaging the mMol/L amount detected in the lemon pectin treatment period divided by the levels in the control period.

## 3. Results

### 3.1. LMW-HDE and HMW-LDE Lemon Pectins Altered Alpha Diversity

**Figure 1:**
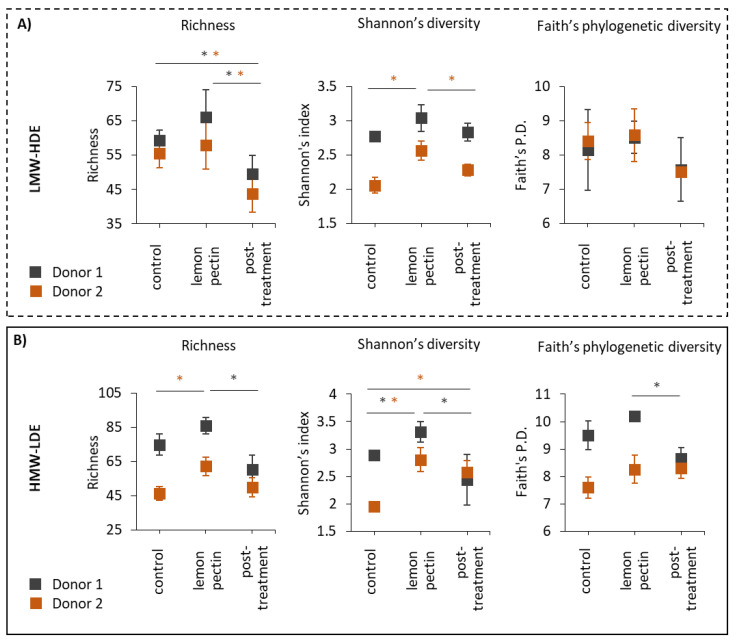
Alpha diversity in terms of richness, Shannon’s diversity, and Faith’s Phylogenetic diversity (Faith’s P.D.). Statistically significant changes are indicated in the figure with an asterisk (*) symbol. (**A**) LMW-HDE lemon pectin; (**B**) HMW-LDE lemon pectin.

To evaluate the effects of low molecular weight, high degree of esterification (LMW-HDE) and high molecular weight, low degree of esterification (HMW-LDE) lemon pectins on the gut microbiota, community structure was analyzed using 16S rRNA gene sequencing. Alpha diversity was calculated during the control, lemon pectin treatment, and post-treatment periods based on richness, which indicated the number of taxonomic units detected, diversity based on Shannon’s diversity index, and biodiversity based on Faith’s phylogenetic diversity (Faith’s P.D.) (Figure 1) [22,23]. For LMW-HDE lemon pectin, both donor communities responded with enhanced richness, although this did not reach significance during the treatment period; However, when treatment with LMW-HDE pectin was halted, there was a significant reduction in richness, even below that of the control period (Figure 1A). Diversity as assessed using the Shannon’s index was only affected by LMW-HDE pectin for donor 2, which indicated that this was a donor-dependent change. A donor-dependent change is specific to a single donor and stems from the fact that the gut microbiota composition is unique to each person, with high inter-individual variability [41]. Faith’s P.D. was not affected by LMW-HDE lemon pectin for either donor. 

For HMW-LDE lemon pectin, both donor communities responded with an increase in richness (Figure 1B). However, this was only significant for donor 2, whereas donor 1 only had a significant decrease in richness in the post-treatment period after pectin administration was halted, similar to what was observed for LMW-HDE pectin. Both donors produced a significant increase in Shannon’s diversity in response to HMW-LDE pectin, and donor 1 had a significant decrease in Faith’s P.D. during the pre-treatment period. Together, these data indicated that both LMW-HDE and HMW-LDE lemon pectins affected community diversity, although a number of these changes were driven by the donor’s individual community composition (Figure 1).

### 3.2. Community Structure Assessed by Principal Coordinates Analysis (PCoA)

**Figure 2:**
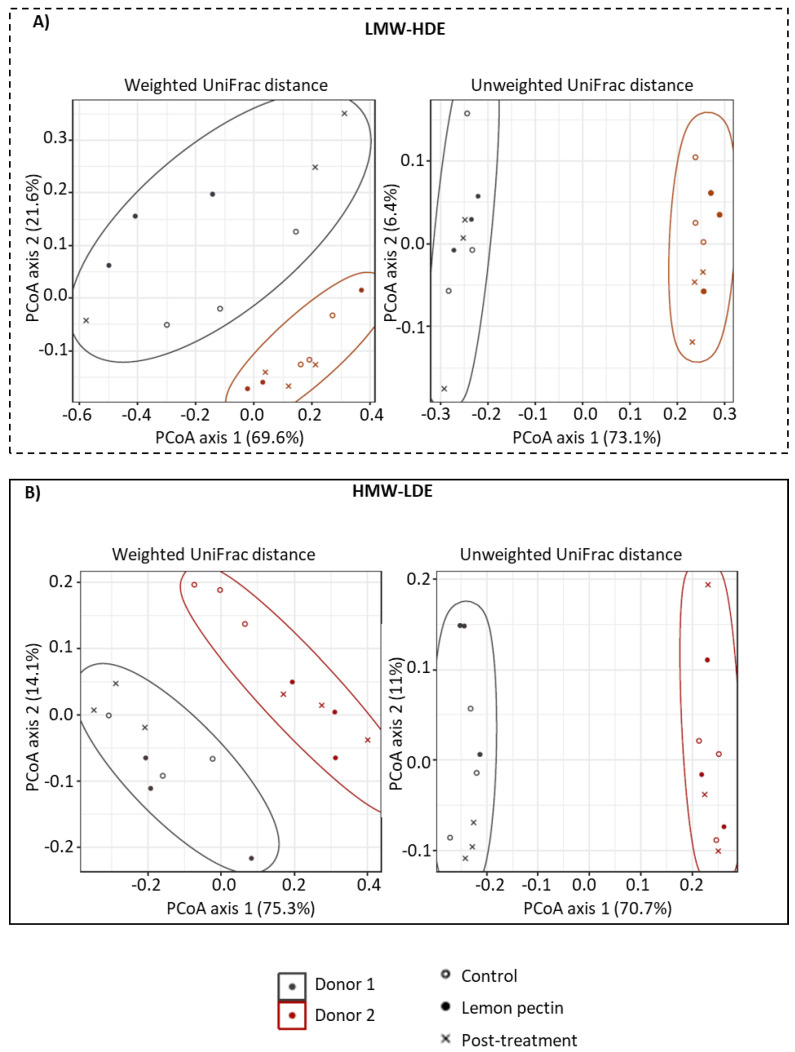
Principal Coordinate analysis (PCoA) based on weighted and unweighted UniFrac distances for both donors during the control, lemon pectin treatment, and post-treatment periods. (**A**) LMW-HDE lemon pectin; (**B**) HMW-LDE lemon pectin.

Next, whether or not treatment with either LMW-HDE or HMW-LDE lemon pectin would affect beta diversity was tested using a principal coordinate analysis (PCoA) based on weighted and unweighted UniFrac distances (Figure 2). This type of analysis provides a visual depiction of the community structures in comparison to each other based on phylogeny. The weighted UniFrac distance considers both the taxa present and their abundance, and the unweighted UniFrac distance considers only the presence or absence of taxa.

In both the weighted and unweighted analyses, the communities for the control, lemon pectin treatment, and post-treatment periods for each donor are clustered together and indicated in the figure with colored circles (Figure 2). The largest variance occurred for the unweighted UniFrac distances in which the PCoA axis 1 was 73.1% for the LMW-HDE lemon pectin and 70.7% for HMW-LDE lemon pectin. This indicated that in both cases, >70% variation among samples appeared to be due to donor origin. This separation depicts a large divergence in community structure in terms of which taxa are present. 

### 3.3. The Structural Impact of Lemon Pectin Based on 16S rRNA Gene Sequencing

**Figure 3:**
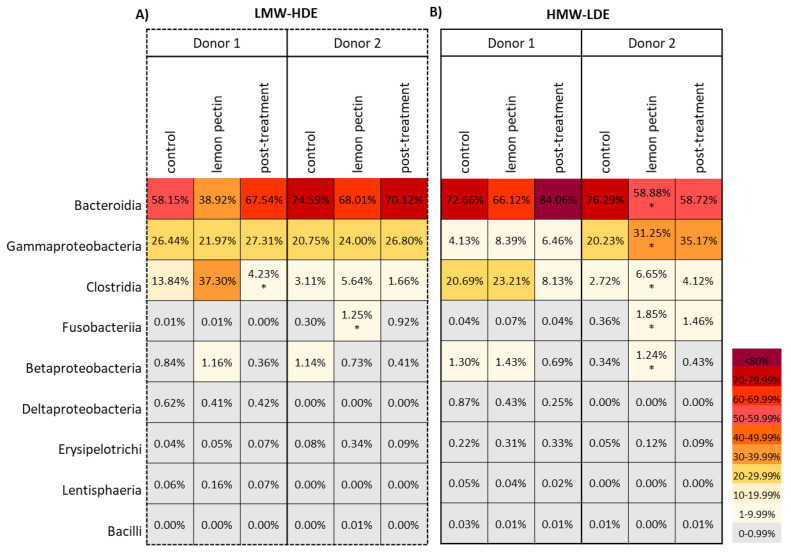
Heatmap depicting relative abundance at the class level for taxa present at >0.01% percent abundance in at least one sample. Significant changes are indicated in the figure with an asterisk (*) symbol. (**A**) LMW-HDE pectin. (**B**) HMW-LDE pectin.

Although the results of PCoA did not show a significant change in community structure, whether or not the addition of LMW-HDE or HMW-LDE lemon pectin would have an impact on specific taxonomic groups was questioned. Using 16S rRNA gene sequencing data, the relative abundance of taxa for each donor during the control, lemon pectin treatment, and post-treatment periods was determined and formatted as heat maps illustrating the community profiles at the class level (Figure 3). Donor 1 communities did not respond significantly during treatment with either pectin; However, there was a significant reduction in class Clostridia and corresponding increase in Bacteroidia post-treatment when LMW-HDE was removed (Figure 3A). It should be noted here that although there was a large increase in Clostridia during lemon pectin treatment this did not reach significance due to variation between samples. Donor 2 communities responded to both types of lemon pectin. There was a significant increase in Fusobacteriia in response to LMW-HDE pectin, and multiple significant changes in response to HMW-LDE treatment, i.e., a decrease in Bacteroidia, and an increase in Gammaproteobacteria, Clostridia, Fusobacteriia, and Betaproteobacteria (Figure 3B).

**Figure 4:**
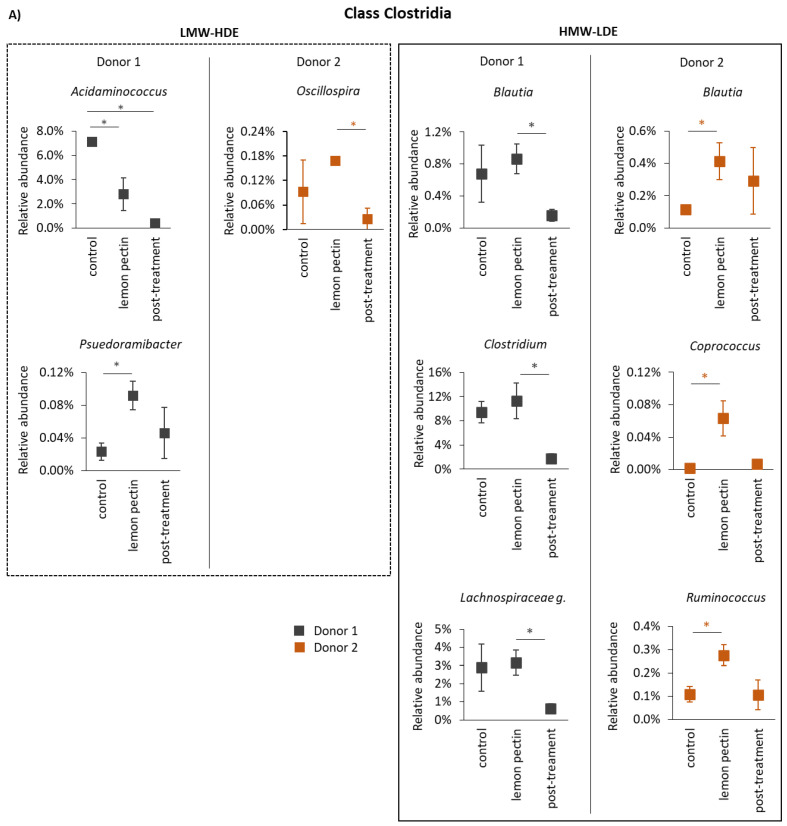

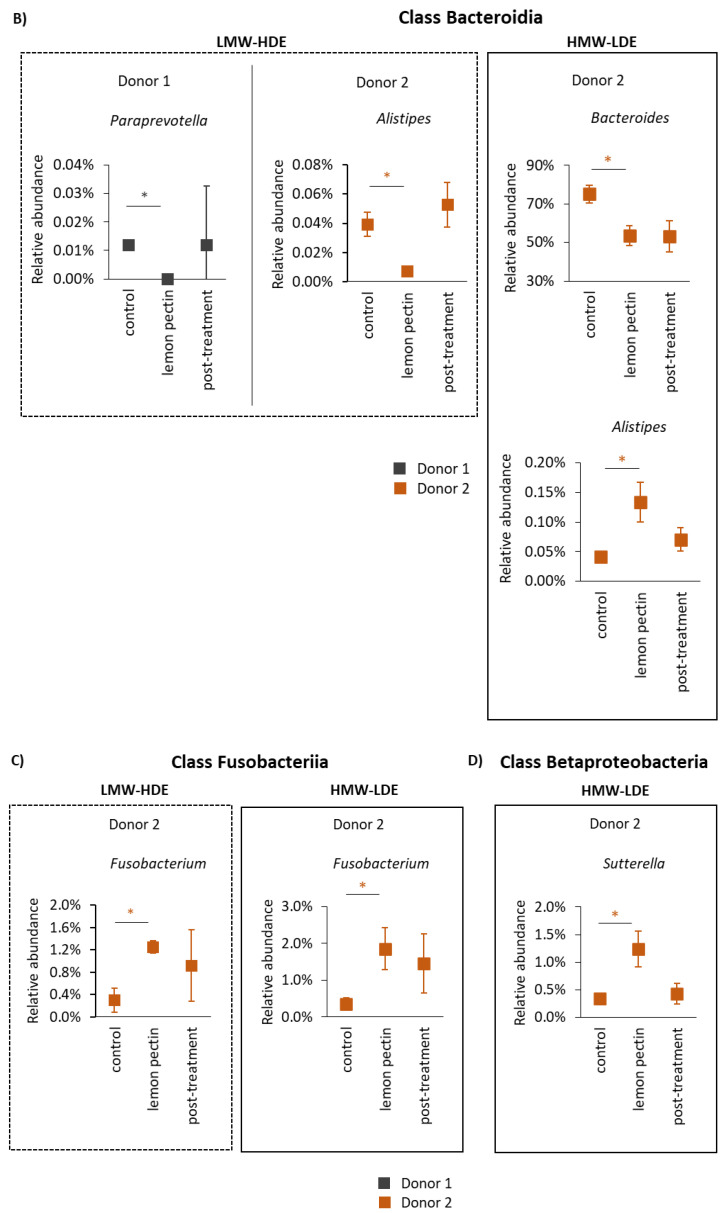
Genus-level taxa significantly altered by LMW-HDE and HMW-LDE lemon pectin. Statistically significant changes are indicated in the figure with an asterick (*) symbol. Taxa from both donors, effected by either the LMW-HDE or the HMW-LDE lemon pectin are grouped according to class. (**A**) Clostridia; (**B**) Bacteroidia; (**C**) Fusobacteriia; (**D**) Betaproteobacteria.

While these results were interesting, it was considered that changes might have occurred at a lower taxonomic level that were missed. Therefore, the statistically significant changes that occurred at the genus level were determined to provide granularity regarding the communities’ response to these lemon pectins (Figure 4). At the genus level, only six taxa were significantly altered by LMW-HDE pectin (Figure 4). This was somewhat unexpected because there was an observed increase in community richness shown in Figure 1, indicating that some taxa that were below the threshold of detection during the control period increased to levels above the limit of detection. Additionally, the taxa responding were not uniform, i.e., the different donor communities responded differentially. For the donor 1 communities, there was a significant decrease in *Acidaminococcus* and *Paraprevotella*, and a corresponding increase in *Psuedoramibacter*. In donor 2 communities, there is a decrease in *Alistipes* and an increase in *Oscillospira* and *Fusobacterium*.

Comparatively, treatment with HMW-LDE lemon pectin produced a larger number of changes, a few of which were donor- independent, or were observed in both donor communities (Figure 4B). In particular, there was a large increase e in taxa from the family *Lachnospiraceae*, although the specific genus level taxa varied between donors. For donor 1, changes to family *Lachnospiraceae* came from the genera *Blautia, Clostridium*, and one unidentified genus, and for donor 2 they came from the genera *Blautia, Coprococcus*, and *Ruminococcus*. For HMW-LDE lemon pectin, there were also a few donor-dependent changes that occurred as well. Interestingly, donor 1 communities only responded with changes in taxa from class *Clostridia*, even though as a whole this class was not altered significantly, as shown in Figure 3. Donor 2 communities had a larger number of taxa respond to HMW-LDE pectin, including an increase in *Alistipes, Sutterella*, and *Fusobacterium*, and a decrease in *Bacteroides*.

**Figure 5:**
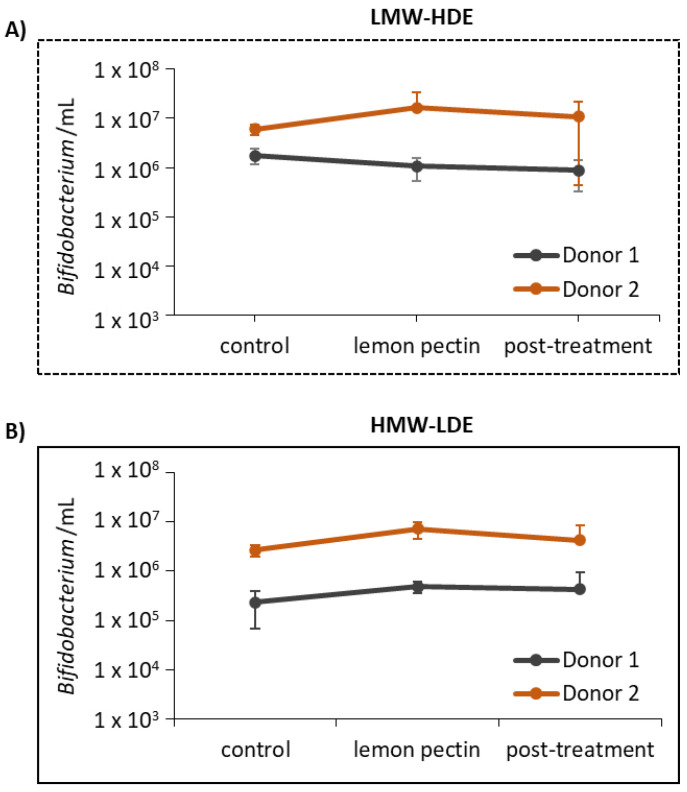
*Bifidobacterium* levels as determined by qPCR analysis. (**A**) LMW-HDE lemon pectin; (**B**) HMW-LDE lemon pectin.

The one taxon that was missing from this community profile was *Bifidobacterium*, which is commonly associated with positive health outcomes and some reports have described pectin as bifidogenic [19]. This absence is likely due to the fact that the *Bifidobacteriales* 16S rRNA gene is not recovered well with the V1-V2 sequencing primers [42]. The 16S rRNA sequencing data was supplemented with qPCR detecting genus *Bifidobacterium* to analyze changes to this taxon (Figure 5). The results found no statistically significant difference in *Bifidobacterium* levels for either donor communities when provided LMW-HDE or HMW-LDE pectin.

### 3.4. Short-Chain Fatty Acid Analysis

**Figure 6:**
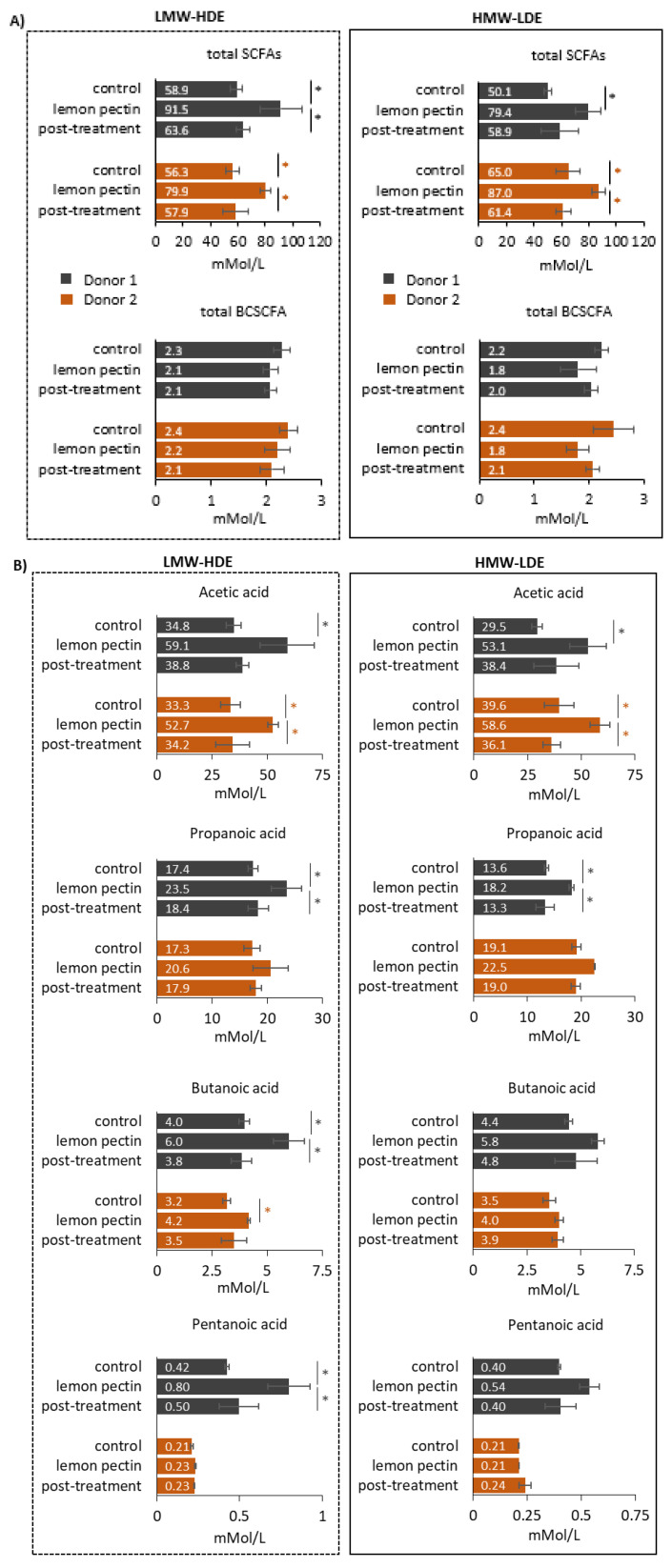
Levels of SCFAs produced during LMW-HDE and HMW-LDE lemon pectin treatment. Statistically significant changes are indicated in the figure with an asterisk (*) sign. (**A**) total SCFAs and total BCSCFAs; (**B**) Levels of individual SCFAs: acetic, propanoic, butanoic, and pentanoic acids.

The gut microbiota is well known for its fermentation properties, and the end result of fermentation is the release of SCFAs, which are typically considered as beneficial to human health [43]. The most prevalent SCFAs are acetic, propanoic, and butanoic acids which are formed from the fermentation of plant polysaccharides, oligosaccharides, and resistant starch [44]. Branched chain SCFAs (BCSCFAs) are the end results of the fermentation of amino acids, proteins, and peptides. To analyze the impact of pectin on the metabolic output of the gut microbiota communities, SCFAs were quantified using GC-MS and amounts produced during the control, lemon pectin treatment, and post-treatment periods were determined and compared (Figure 6).

The results showed that the levels of total SCFAs were significantly increased in response to both LMW-HDE and HMW-LDE pectin, and this occurred for both donor communities (Figure 6A). For LMW-HDE there was an average 1.49-fold increase and for HMW-LDE there was an average 1.46-fold increase in total SCFA levels. Neither lemon pectin influenced levels of BCSCFAs for either donor. 

When evaluated more in-depth, it was discovered that the increase in total SCFAs was largely due to significantly increased levels of acetic acid, which was the predominant SCFA measured in these communities (Figure 6B). This significant increase was observed for both treatment conditions and was donor independent. Treatment with either-LMW-HDE or HMW-LDE lemon pectin resulted in an average 1.64-fold increase in acetic acid when compared to the control period. Specifically, for LMW-HDE lemon pectin treatment, the increase in acetic acid corresponded with a significant increase in butanoic acid for both donors, with an average increase of 1.41-fold compared to the control period. Interestingly, donor 1 responded to both lemon with an additional significant increase in propanoic acid, and LMW-HDE lemon pectin further increased levels of pentanoic acid for this donor community. 

## 4. Discussion

The prebiotic function of pectin has been described in previous studies that have demonstrated its ability to promote the growth of beneficial bacteria [9,45,46,47]. Yet, the results in the literature have been somewhat conflicting, most likely due to the difference in chemical structure of the pectins used [9]. The MW and DE of pectin influences its fermentability by the gut microbiota and has an effect on its prebiotic potential. Therefore, a better understanding on how these structural components of pectin influence its ability to affect the gut microbiota was warranted. It should be noted that based on the experimental design of this study, it is impossible to determine whether the observed outcomes were due to the lemon pectin MW or DE, or a combination of these two properties. Future work will aim at elucidating the role of lemon pectin MW and DE specifically on its utilization by the gut microbiota and its ability to alter structure and function of this community. Here, the goal was to see the maximal divergence in prebiotic effect that would occur from two lemon pectins that were structurally distinct in both terms of MW and DE. In this study, two lemon pectins of different MW and DE were tested on the gut microbiota of two donors using an in vitro culturing strategy. Changes that occurred to the community structure and function were evaluated in order to determine their prebiotic effects and compare how the different structures may elicit alternate responses. 

The results of this study showed that both LMW-HDE and HMW-LDE lemon pectins were able to produce changes to the gut microbiota community structure, although the type of changes observed were variable and many were donor-dependent or were reliant on the composition of the individual donor’s community. In terms of alpha diversity, here, richness was enhanced by both lemon pectins, although this did not reach statistical significance for both donors and was more apparent for HMW-LDE lemon pectin (Figure 1). These results support the findings from previous studies that reported pectin treatment increased community richness and contradict those that have found alpha diversity decreased with pectin treatment [9,48,49]. However, these discrepancies are most likely due to the different types of pectins utilized and the study design. 

An underlaying observation for community structure in this study was that the effect of both LMW-HDE and HMW-LDE lemon pectins were highly contingent on the composition of the individual donor communities. This was most apparent in the results of the weighted and unweighted UniFrac analyses, which showed that for both the LMW-HDE and the HMW-LDE lemon pectins the largest divergence was due to donor composition and not pectin treatment (Figure 2). Yet, this was expected since these two communities originated from different donors. However, within each donor, there was no determinable pattern of community change when either of the lemon pectins was added or removed. Instead, the lack of clustering by treatment type indicated that adding 1% lemon pectin, either the LMW-HDE or the HMW-LDE, was insufficient to elicit detectable changes in the community structure that were visible using PCoA. The donor-dependent effect was also observed in community profiles examined at the class level (Figure 3). In this heat map, it was apparent that there were statistically significant changes occurring for both LMW-HDE and HMW-LDE lemon pectins, but they were donor-dependent, indicating again that the changes elicited by these pectins relied on the composition of the starting communities. 

When the community structure was analyzed at higher resolution, looking at the genus level, LMW-HDE lemon pectin affected levels of multiple taxa, yet these were still considered as donor-dependent (Figure 4). Alternatively, HMW-LDE lemon pectin produced significant changes in genera within family *Lachnospiraceae* that occurred in both donor communities and was therefore not donor-dependent. This observation aligns with a previous report that found pectin treatment to increase levels of *Lachnospiraceae*, which contains some of the enzymes required to degrade the pectin structure [10]. However, it was also observed that Donor 2 communities had a larger number of taxa respond to HMW-LDE pectin, including an increase in *Alistipes, Sutterella*, and *Fusobacterium*, and a decrease in *Bacteroides*. These changes were remarkable because when donor 2 communities were treated with LMW-HDE pectin, there was a decrease in *Alistipes*, whereas the HMW-LDE pectin enhanced levels of this taxa. The decrease in *Bacteroides* was unexpected, as many members of this genus carry enzymes capable of performing pectin fermentation [8,9]. While *Alistipes*, as a member of the *Bacteroides*, may be pectinolytic, increases in the non-pectinolytic *Sutterella* have also been observed with pectin additions, with the latter being attributed to cross-feeding. Thus, the responses observed here are likely inclusive of both direct and indirect metabolic benefit from the pectin [6,50].

Several previous studies have reported the bifidogenic effects of pectin [16,20]. Based on these reports, the ability for LMW-HDE and HMW-LDE lemon pectins to effect *Bifidobacterium* was assessed using qPCR. Here, neither lemon pectin significantly altered *Bifidobacterium* levels for either donor (Figure 5). However, this may be due to the in vitro design for this experiment, as this culturing method may not promote the maintenance or growth of *Bifidobacterium*, or *Bifidobacterium* may be unable to properly utilize lemon pectin or cross-feed with taxa capable of metabolizing these pectin structures. Taken together, results generated from 16S sequencing data and qPCR quantification provided some interesting, yet conflicting results. The addition of lemon pectin elicited changes to the community structure, yet these were somewhat variable and depended on the donor-specific communities, and did not necessarily reproduce results as previously reported, i.e., no bifidogenic response [19]. However, this may have been due to the low amount of pectin used in this study (1%), or it is possible that lemon pectin is not specifically targeting any one taxon within the community but is able to be utilized by a number of taxa. 

Next, the ability for LMW-HDE and HMW-LDE lemon pectins to alter community function was analyzed based on SCFA quantification (Figure 6). Here, a significant increase in levels of total SCFAs was observed for both lemon pectins in a donor-independent manner. This indicated that fermentation occurred, regardless of the MW and DE of the two lemon pectins. This was expected since it is known that the gut microbiota can metabolize pectin and produce SCFAs, and these results confirm a previous report that found MW and DE did not impact SCFA production [19]. There were no significant changes to levels of total BCSCFA, which was also expected, as pectin is not involved in protein or amino acid metabolism. 

Interestingly, the results showed that while both lemon pectins enhanced total levels of SCFAS, they did so through the promotion of different types of SCFAs. This supported previous findings on total levels SCFAs [19] and adds to the current knowledge on how pectin structure dictates its prebiotic potential. Here, LMW-HDE lemon pectin increased levels of acetic and butanoic acids, whereas HMW-LDE lemon pectin only stimulated acetic acid. Both sets of observations reached statistical significance in a donor-independent manner. These results indicated that while the community structure may have changed in a donor-dependent manner, the effect on SCFA production is not, and the MW and DE of lemon pectin determined which types of SCFAs increased, with LMW-HDE lemon pectin favoring an increase in acetic and butanoic acids, verses HMW-LDE lemon pectin favoring an increase in acetic acid. This information is valuable for those selecting a prebiotic based on its ability to enhance specific types of SCFAs. 

The production of SCFAs through fermentation is an extremely important factor to consider in gut microbiota studies because they can be metabolized by the cells of the GIT or can be taken up and circulated throughout the body via the bloodstream. Butanoic acid is particularly important for the colonic epithelium as its oxidation supplies approximately 70% of the energy needed for the cells there [51]. Acetic, propanoic, and butanoic promote cellular turnover and differentiation in the GIT which aids in wound healing and promotes strong barrier formation [52,53]. These functions can be attributed to SCFAs ability to function as histone deacetylase inhibitors, predominantly propanoic and butanoic acid, and as activators of G-protein-coupled receptors (GPCRs) [54]. GPR41 and GPR43 are two free fatty acid receptors known to be activated by fatty acids with less than six carbons. In the intestine, sympathetic nervous system and adipose tissue these receptors serve as energy sensors and regulators in response to propionic acid and acetic acid, respectively [55,56]. SCFAs are also known to mediate inflammatory response through these GPCRs, but allergic response elicited by pentanoic acid, in keratinocytes appears to be dependent on a Gq/11-coupled protein receptor that is not GPR41 or GPR43. This suggests that SCFAs may have yet more unidentified receptors within the body [57]. The differential release of SCFAs for LMW-HDE and HMW-LDE lemon pectin may promote cellular health in slightly different, but complementary ways.

## 5. Conclusions

In conclusion, the results of this study demonstrated that lemon pectins of varying MW and DE had differential effects on the gut microbiota structure and function. For LMW-HDE lemon pectin, community structure was altered in a donor-dependent manner, with changes noted in *Acidaminococcus*, *Paraprevotella*, *Psuedoramibacter*, *Alistipes, Oscillospira,* and *Fusobacterium*. For HMW-HDE lemon pectin, there were donor-independent shift in taxa within *Lachnospiraceae*. When looking at function, the data showed a donor-independent enhancement of SCFA production for both LMW-HDE and HMW-LDE lemon pectin. This was predominantly from acetic acid which increased 1.64-fold during the treatment period for both lemon pectins used. However, treatment with LMW-HDE lemon pectin also increased butanoic acid by 1.41-fold, which was a novel finding. This data provides further evidence that pectin structure influences its ability to be utilized by the gut microbiota, which directly relates its potential prebiotic effect.

## Data Availability

Data will be made available following review.

## Supplementary Materials

The following supporting information can be downloaded at: https://www.mdpi.com/article/10.3390/foods11233877/s1, Figure S1. Experimental design; Figure S2. Donor inoculum composition; Table S1. Lemon pectin specifications.
