# Supplementary material for: Modulation of the Gut Microbiota Structure and Function by Two Structurally Different Lemon Pectins
Source: Foods. 2022 Dec 1;11(23):3877. doi: 10.3390/foods11233877 (PMC9739951; doi:10.3390/foods11233877)
Supplement: Supplementary file 1 [file foods-11-03877-s001.zip › foods-2000852-supplementary.pdf]

Supplementary Figure S1.

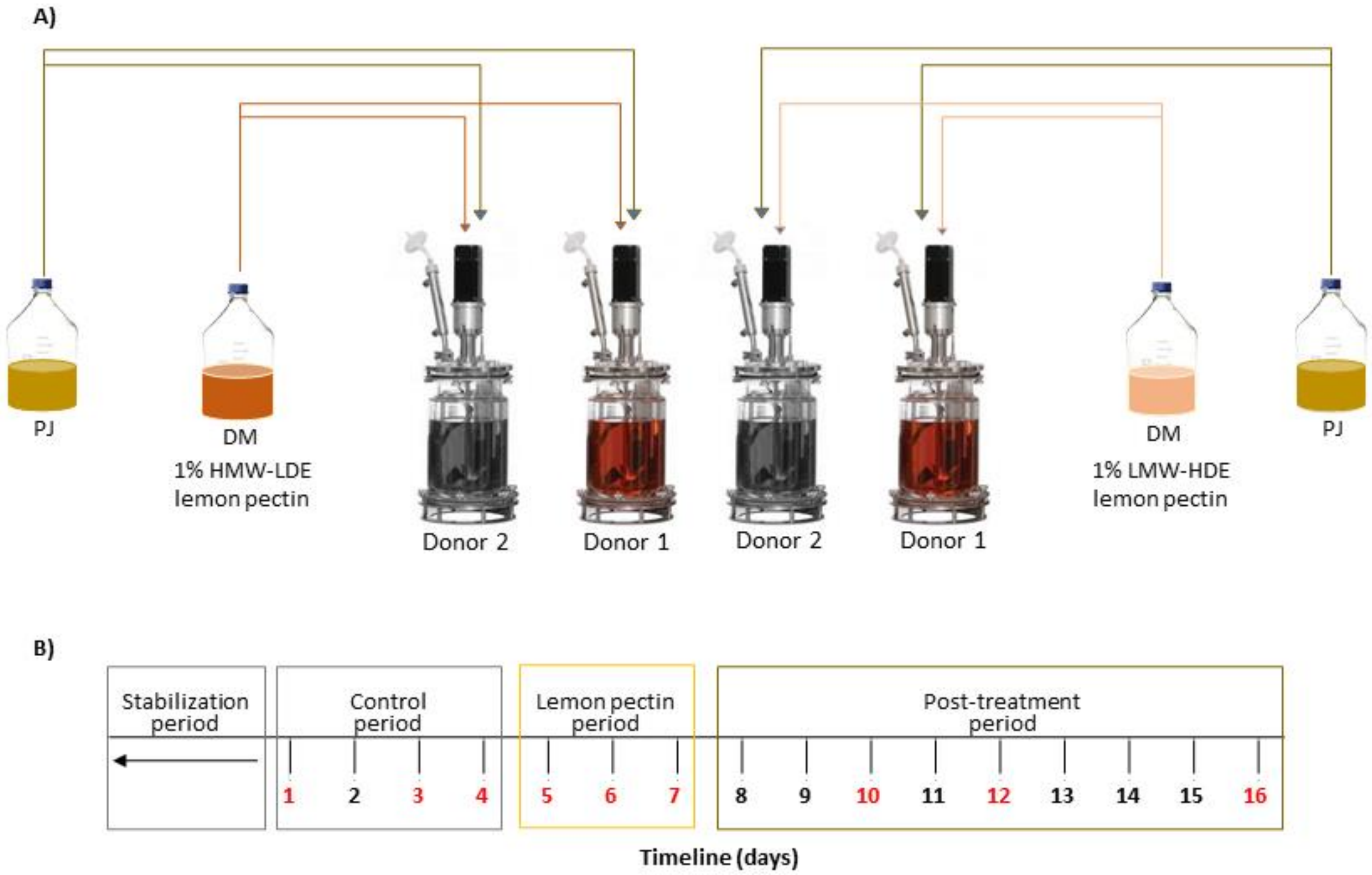

Supplementary Figure S1. Experimental design. A) Schematic of experimental set-up. B) Timeline for the experiment.

Supplementary Figure S2.

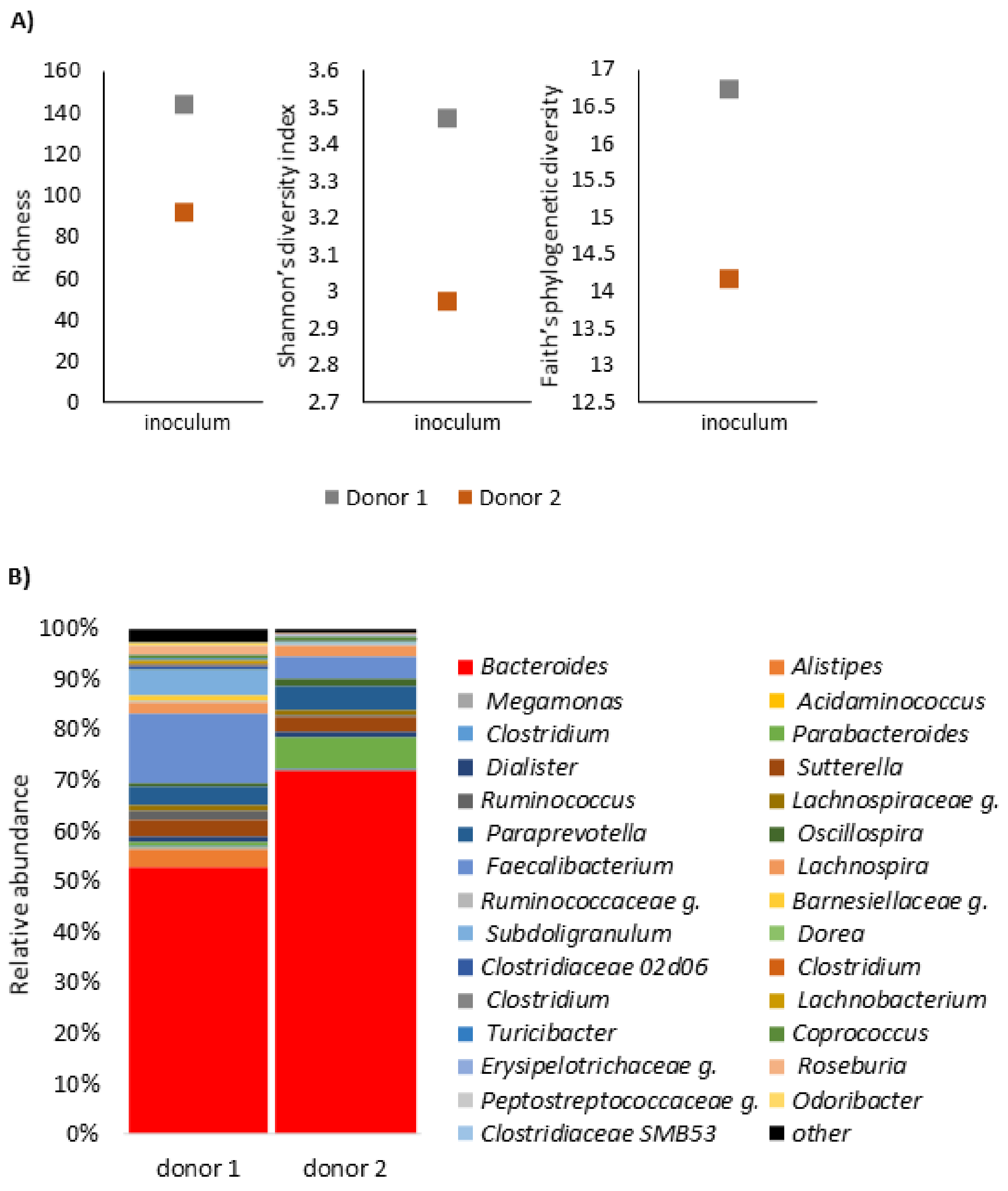

Supplementary Figure S2. Taxonomic composition of the fecal homogenates used for *in vitro* cultivation. A) Alpha diversity of each inoculum based on richness, Shannon's diversity index, and Faith's phylogenetic diversity. B) Relative abundance of genera present at greater than 0.1% abundance based on 16S rRNA gene sequencing.

Supplementary Table S1.

	M_w, kDa*	GA, %**	DE, %***
Lemon pectin 1 (LMW-HDE)	122	64.7	66
Lemon pectin 2 (HMW-LDE)	308	69.0	31

* The average molar weight
** Galacturonic acid content
*** Degree of esterification
